# UPS-indel: a Universal Positioning System for Indels

**DOI:** 10.1038/s41598-017-14400-1

**Published:** 2017-10-26

**Authors:** Mohammad Shabbir Hasan, Xiaowei Wu, Layne T. Watson, Liqing Zhang

**Affiliations:** 1 Department of Computer Science, Virginia Tech, Blacksburg, VA 24061 USA; 2 Department of Statistics, Virginia Tech, Blacksburg, VA 24061 USA; 3 Department of Mathematics, Virginia Tech, Blacksburg, VA 24061 USA; 4 Department of Aerospace and Ocean Engineering, Virginia Tech, Blacksburg, VA 24061 USA

## Abstract

Storing biologically equivalent indels as distinct entries in databases causes data redundancy, and misleads downstream analysis. It is thus desirable to have a unified system for identifying and representing equivalent indels. Moreover, a unified system is also desirable to compare the indel calling results produced by different tools. This paper describes UPS-indel, a utility tool that creates a universal positioning system for indels so that equivalent indels can be uniquely determined by their coordinates in the new system, which also can be used to compare different indel calling results. UPS-indel identifies 15% redundant indels in dbSNP, 29% in COSMIC coding, and 13% in COSMIC noncoding datasets across all human chromosomes, higher than previously reported. Comparing the performance of UPS-indel with existing variant normalization tools vt normalize, BCFtools, and GATK LeftAlignAndTrimVariants shows that UPS-indel is able to identify 456,352 more redundant indels in dbSNP; 2,118 more in COSMIC coding, and 553 more in COSMIC noncoding indel dataset in addition to the ones reported jointly by these tools. Moreover, comparing UPS-indel to state-of-the-art approaches for indel call set comparison demonstrates its clear superiority in finding common indels among call sets. UPS-indel is theoretically proven to find all equivalent indels, and thus exhaustive.

## Introduction

Indel stands for insertion or deletion of bases in a DNA sequence. As the second most common form of genetic variation, indels play an important role in genome and protein evolution. Due to artificial factors such as sequencing errors, ambiguous alignment of the reads, inconsistent ways of representing the same variant by different tools, the same mutation may be recognized as distinct variations occurring at different locations^1–3^. For example, consider a reference sequence AGGAAAGAAAGAAAGAAAGAG ranging from position 100285630 to 100285650 and two indels stored in dbSNP, rs147659011 (GAAA/+) and rs60376183 (AAGA/+), annotated to this region with positions 100285632 and 100285650, respectively. Although these indel mutations may indeed occur at different positions, they are biologically equivalent because they result in the same altered sequence AGGAAAGAAAGAAAGAAAGAAAGAG. Supplementary Table 1 shows another example of redundant insertion and deletion in dbSNP. Since many databases such as dbSNP, Database of Genomic Variants (DGV), and Ensembl combine indels resulting from large-scale studies, similar cases often exist in those databases, leading to a nonnegligible problem of data redundancy. In fact, about 10%^4^ of the human indels stored in dbSNP and 18%^1^ in Ensembl are redundant. Resolving the indel redundancy in major databases is important for subsequent genetics research. Nevertheless, this problem has not been given the attention it deserves.

Numerous approaches have been developed for systematic comparison of indels to determine equivalence and hence solve the redundancy problem. The “strict matching” approach matches two indels if they share the same position, reference, and alternate alleles in two different entries in the VCF file. However, as demonstrated in ref.^3^, this approach fails to find equivalent indels that are not identical. The “distance based approach” treats two indels as equivalent if both have the same length and occur within a certain distance such as ±5 bp^5^ or ±25 bp^6^. However, this approach introduces false positives when neighboring indels are not equivalent^1^ and misses equivalent indels that are farther apart than the distance cutoff. Clearly, selection of an optimal distance cutoff is a tradeoff of the two types of errors: smaller distance cutoffs result in a decreased false positive rate but an increased false negative rate.

To address the limitations of the two aforementioned approaches, the more widely used “normalization” approach attempts to solve the indel redundancy problem by left (or right) normalization, i.e., consistently shifting the start position of an indel to the left (or right) as long as the resulting sequence is the same as the one generated by the original mutation^7^. Tools using this type of variant normalization include vt normalize^2^, BCFtools^8^, and GATK LeftAlignAndTrimVariants^9^. These tools usually take a VCF file as input, output another VCF file with canonical VCF entries for the indels after normalization, and then perform “strict matching” to find equivalent indels with exactly the same canonical representation. The normalization approach generally performs well in identifying equivalent indels, but as shown in the result section, fails to normalize complex variants.

The positions of indels may get changed after left/right normalization, potentially misleading downstream analysis. For example, the deletion rs536379477 resides in the exon of the transcript ENST00000590192.1, but the equivalent deletion rs41436444 is in the intron of the same transcript. Therefore reporting these two indels with the same normalized position might lead to missing significant insight into genetic diseases or phenotypes of interest. Since the exact positions of most indel variations are not known, it is thus best to represent the indel of interest with a range of positions, within which equivalent indels can occur, rather than as a single normalized position. A similar idea was proposed by Krawitz *et al*.^10^.

This paper proposes UPS-indel, a universal positioning system for indels, whereby every indel variant is represented by a range of positions within which all equivalent indels can occur. This representation is added to the VCF file resulting in a UVCF file containing not only the original indel calling results, but also the complete representation of all equivalent indels. The advantage of adding this column of information to the existing VCF file is (1) the original VCF file structure is unchanged so the UVCF file is still compatible with many downstream programs, (2) the UPS-indel notation facilitates the comparison of indels from different VCF files, (3) for equivalent indels that overlap both coding and noncoding regions, having the range column in the indel calling output would allow a downstream indel annotation system to consider the range rather than a single position, possibly annotating both a coding and noncoding variant. In summary, this work extends the previous work of Krawitz *et al*.^10^ and Assmus *et al*.^1^ by a new coordinate system Universal Positioning System (UPS), a rigorous mathematical proof that all (deletion and insertion) equivalent indels are found, the handling of complex variants, and a simple modification of an input VCF file to produce an output UVCF file containing the indel equivalence information. Results show that UPS-indel identifies more redundant indels than the existing approaches, also enables a comparison between indel calling results produced by different indel callers, and performs better than other state-of-the-art approaches for finding indels in common among call sets.

## Materials and Methods

This section defines some terms frequently used in this paper.

### Alternate Sequence

A sequence that is produced by introducing a specific indel to the reference sequence at a specific position. This is also known as the *mutant sequence*.

Let *R* be the reference sequence and *p* be either an insertion or a deletion of a given length that occurs at a given position in the reference sequence. The alternate sequence for insertion is denoted by *R*′_*I*_ = *R* + *p* and for deletion by *R*′_*D*_ = *R* − *p*.

### Equivalent Indels

Two indels are considered equivalent if and only if they produce the same alternate sequence. Note that equivalent indels must be of the same type (insertion and deletion) and same length.

### Redundant Indels

Equivalent indels that are reported as distinct entries in a VCF file are defined as redundant indels.

### Region of Equivalence

This is defined as the range of positions in the reference sequence where equivalent indels occur.

### Cyclic Permutation

A permutation (*y*_*0*_, *y*_1_, *y*_2_, …, *y*_n−1_) = *f*(*x*_*0*_, *x*_1_, *x*_2_, …, *x*_n−1_) where *y*_*i*_ = *x*_(*i*+*k*)*mod* *n*_ for 0 ≤ *i* ≤ *n* *−* 1, *k* can be positive (left cyclic) or negative (right cyclic). For example: for a string “ATCG”, the left cyclic permutations are TCGA, CGAT, and GATC; the right cyclic permutations of this string are GATC, CGAT, and TCGA.

Table 1 shows an example of equivalent indels. Observe that all equivalent indels are cyclic permutations of each other (e.g., a cyclic permutation of CT is TC and cyclic permutations of TGT are GTT and TTG) and equivalence continues until there is a mismatch (see Supplementary Table 2). This observation leads to the following theorem.

**Table 1:** An example of equivalent indels.

Equivalent insertions		Equivalent deletions	
Reference, *R*	GTCTA	Reference, *R*	ACTGTTGTG
Case 1, *R*′_*I*_	G[TC/+]TCTA	Case 1, *R*′_*D*_	AC[TGT/−]TGTG
Case 2, *R*′_*I*_	GT[CT/+]CTA	Case 2, *R*′_*D*_	ACT[GTT/−]GTG
Case 3, *R*′_*I*_	GTC[TC/+]TA	Case 3, *R*′_*D*_	ACTG[TTG/−]TG
Case 4, *R*′_*I*_	GTCT[CT/+]A	Case 4, *R*′_*D*_	ACTGT[TGT/−]G

**Theorem 1:** All equivalent indels in the region of equivalence are cyclic permutations of each other.

**Proof:** Consider two equivalent indels *d*_*1*_ and *d*_*2*_ and the equivalence region *R* they define.

For insertion within *R*, the alternate sequences are

*d*_*1*_ *S* = *Sd*_*2*_

for some nonempty *S*. For deletion within *R*, the alternate (possibly empty) sequence is *S* starting with *d*_*1*_ *S* = *Sd*_*2*_.

**Case 1**. For *|S*| < *|d*_*1*_|, *d*_*1*_ = *SX* for nonempty *X* and *d*_*1*_ *S* = *SXS* = *Sd*_*2*_ implies *d*_*2*_ = *XS* is a cyclic permutation of *d*_*1*_ = *SX*.

**Case 2**. For *|S*| = *|d*_*1*_|, *d*_*1*_ = *d*_*2*_ = *S*.

**Case 3**. For *|S*| > *|d*_*1*_|, *S* = *d*_*1*_ *X* for nonempty *X* with |*X*| < *|S*|, and *d*_*1*_ *d*_*1*_ *X* = *d*_*1*_ *S* = *Sd*_*2*_ = *d*_*1*_ *Xd*_*2*_ implies *d*_*1*_ *X* = *Xd*_*2*_. Repeating this argument for *d*_*1*_ *X* = *Xd*_*2*_ eventually reduces *X* to one of the previous two cases.

Another case for deletion is when *R* is periodic with period |*d*_*1*_|, having the form

*R* = *d*_*1*_ *d*_*1*_…….. *d*_*1*_ (*d*_*1*_)_1_ where (*d*_*1*_)_1_ is the first symbol of *d*_*1*_. Then every consecutive subsequence *d*_*2*_ of *R* with |*d*_*1*_| = |*d*_*2*_| is an equivalent deletion, and *d*_*2*_ is a cyclic permutation of *d*_*1*_. **(Q.E.D)**.

**Corollary**. For *|S*| > *|d*_*1*_|, *S* must have the form *d*_*1*_ *d*_*1*_…….*…… *d*_*2*_ *d*_*2*_ with an equal number of *d*_*1*_s and *d*_2_s.

**Figure 1:**
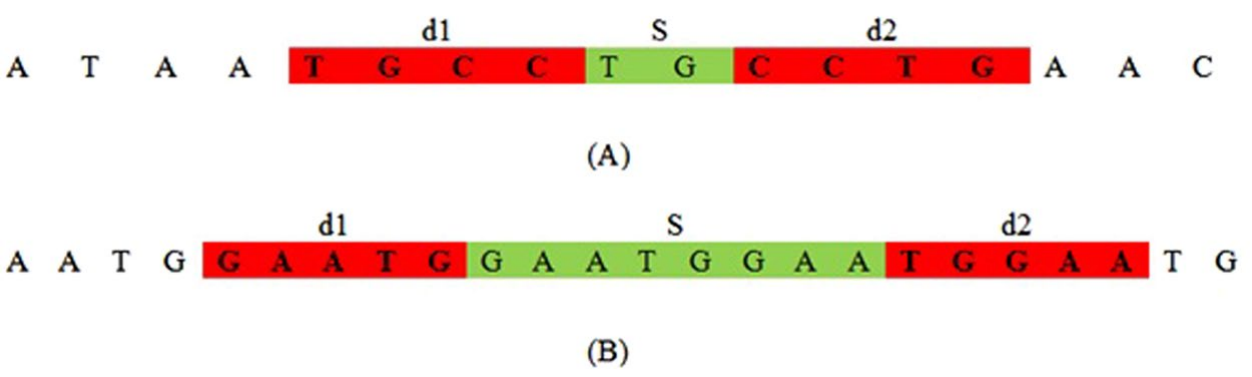
Illustration of two cases of Theorem 1. (A): |*d*_*1*_| > |*S*|, (B): |*d*_*1*_| < |*S*|.

**Figure 2:**
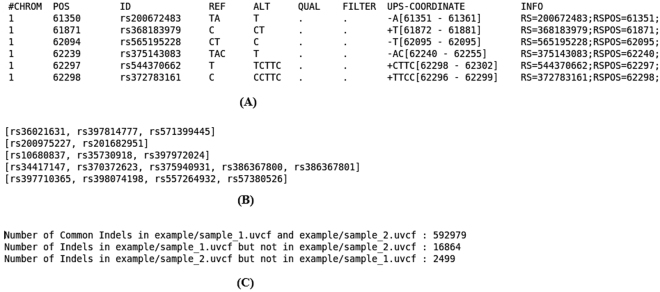
Different utilities of UPS-indel. (A)UVCF format, (B) redundant indel list, and (C) comparing two uvcf files.

Figure 1 illustrates Theorem 1 with two examples. Based on the theorem, an algorithm called UPS-indel (see Table 2) exhaustively increases the range of equivalence as far as possible in both left and right directions from a given indel position. Finally for each indel in the VCF file, the algorithm reports its range of equivalence, which is called the Universal Positioning System coordinate (UPS-coordinate). Once indels are represented by their UPS-coordinates, identifying redundant indels becomes a trivial task of string comparison (e.g., Fig. 2(A), comparison across the 8^th^ column). Note that since UPS-indel implements Theorem 1, which characterizes indels within an equivalence region, UPS-indel is exhaustive, finding all equivalent indels.

**Table 2:** UPS-indel algorithm.

UPS-indel(list_of_indels_in_VCF_file, reference_sequence)
{
For each indel in the list
1. Extract REF allele and ALT allele from VCF file
2. pattern ← diff(REF, ALT)
3. indel ← pattern
4. eq_indel ← getCyclicPermutationFromLeft(indel)
5. pos ← position of indel according to the VCF file
6. position ← pos
7. upperBound ← position
8. str ← reference_sequence (position + 1)
9. while((indel + str) = = (str + eq_indel))
10. indel ← eq_indel
11. upperBound++
12. str ← reference_sequence (position + 1)
13. eq_indel ← getCyclicPermutationFromLeft(indel)
14. End while
15. indel ← pattern
16. eq_indel ← getCyclicPermutationFromRight(indel)
17. position ← pos
18. lowerBound ← position
19. str ← reference_sequence (position - 1)
20. while((str + indel) = = (eq_indel + str))
21. indel ← eq_indel
22. lowerBound–
23. str ← reference_sequence (position - 1)
24. eq_indel ← getCyclicPermutationFromRight(indel)
25. End while
26. if (pattern is an insertion)
27. UPS-coordinate ← + pattern[lowerBound, upperBound]
28. else //pattern is a deletion
29. UPS-coordinate ← –pattern[lowerBound, upperBound]
30. End for
}

Note that “left” and “right” cyclic permutations are equivalent – there is no difference. In line 2 of the UPS-indel algorithm (Table 2), while extracting the “pattern” from the entries of the RFE and ALT columns of the input VCF file, UPS-indel performs horizontal decompositions of the complex variants and assigns the indel part as the value of pattern. For example, suppose in the REF column of a VCF entry there is an allele “ATAA” and in the ALT column there is an allele “AG”. In this case, UPS-indel performs horizontal decompositions of the complex variants to produce two separate entries (AT → AG and AA →*meaning that there is a deletion of AA).

UPS-indel is written in C++ and can run on Linux, Windows, or Mac operating systems that have a C++ compiler. The command line version of UPS-indel is available at https://github.com/shabbir005/ups-indel. UPS-indel uses SeqAn, an open source C++ library containing efficient algorithms and data structures to analyze large genome sequences^11^. The input to UPS-indel is a reference chromosome sequence, a VCF file containing a list of indels, an output file name, and a flag to enable/disable horizontal decomposition, for example,

./ups_indel example/chr1.fa example/chr1.vcf example/chr1.uvcf –hd=true.

This command line produces an output file named chr1.uvcf, containing the UPS-coordinates of all the indels in chr1.vcf when horizontal decomposition is enabled. Figure 2(A) shows an example UVCF file.

The UVCF file keeps the same content/format as the VCF file, with an additional column that contains the indel’s UPS-coordinate information. The interpretation of the UPS-coordinate follows:

- Symbols + and − denote insertion and deletion, respectively, followed by the base pairs inserted/deleted from the reference, and the UPS-coordinate (in square brackets).
- The UPS-coordinate contains a range of positions in the square brackets representing the region of equivalence for the indel. For example, the UPS-coordinate + CTTC [62298 − 62302] means there is an insertion of CTTC at position 62298, and the same alternate sequence can be produced by inserting TTCC at position 62299, or TCCT at position 62300, and so on.

Once indels are represented by the coordinates produced by UPS-indel, one can easily identify redundant indels within one indel call set or multiple indel call sets. For example, the following command line

./ups_generate_redundant_indel_list example/chr1.uvcf example/redundant_indel_list.txt

produces a list of indel groups containing dbSNP IDs of redundant indels (Fig. 2(B)).

UPS-indel groups all redundant indels together. For example, consider a group [rs34748242, rs59148039] with the UVCF entry shown in Table 3. These two indels belong to the same indel type (insertion), have same base pairs inserted (TG), and share the same UPS-coordinate and hence they are considered as equivalent.

**Table 3:** UVCF file for redundant indels.

#CHRM	POS	ID	REF	ALT	QUAL	FILTER	UPS-COORDINATE
1	10009638	rs34748242	T	TTG	.	.	+ TG[10009639 – 10009648]
1	10009639	rs59148039	T	TGT	.	.	+ TG[10009639 – 10009648]

UPS-indel can compare multiple indel call sets. This utility is particularly useful for generating a high-confidence indel call set by taking the intersection of the results of different indel callers^12^, or merging the indel calling results from different tools for a consensus variant caller^13^, or comparing indel call sets generated by different indel callers to determine their relative recall, precision, and accuracy, and to understand the source of their dissimilarities. To use this utility of UPS-indel, after converting two VCF files to UVCF files, one can use the following command to get the comparison result (Fig. 2(C)), which contains useful statistics for downstream analysis:./ups_compare_uvcf_files example/sample1.uvcf example/sample2.uvcf example/comparison_result.txt

A utility for UPS-indel can produce a filtered UVCF file after removing redundant indels. The following command, for example, can get the filtered UVCF file named out_filtered.uvcf containing nonredundant indels:

java GenerateFilteredUVCFFileAfterRemovingRedundantIndel example/out.uvcf example/redundant_indel_list.txt

**Figure 3:**
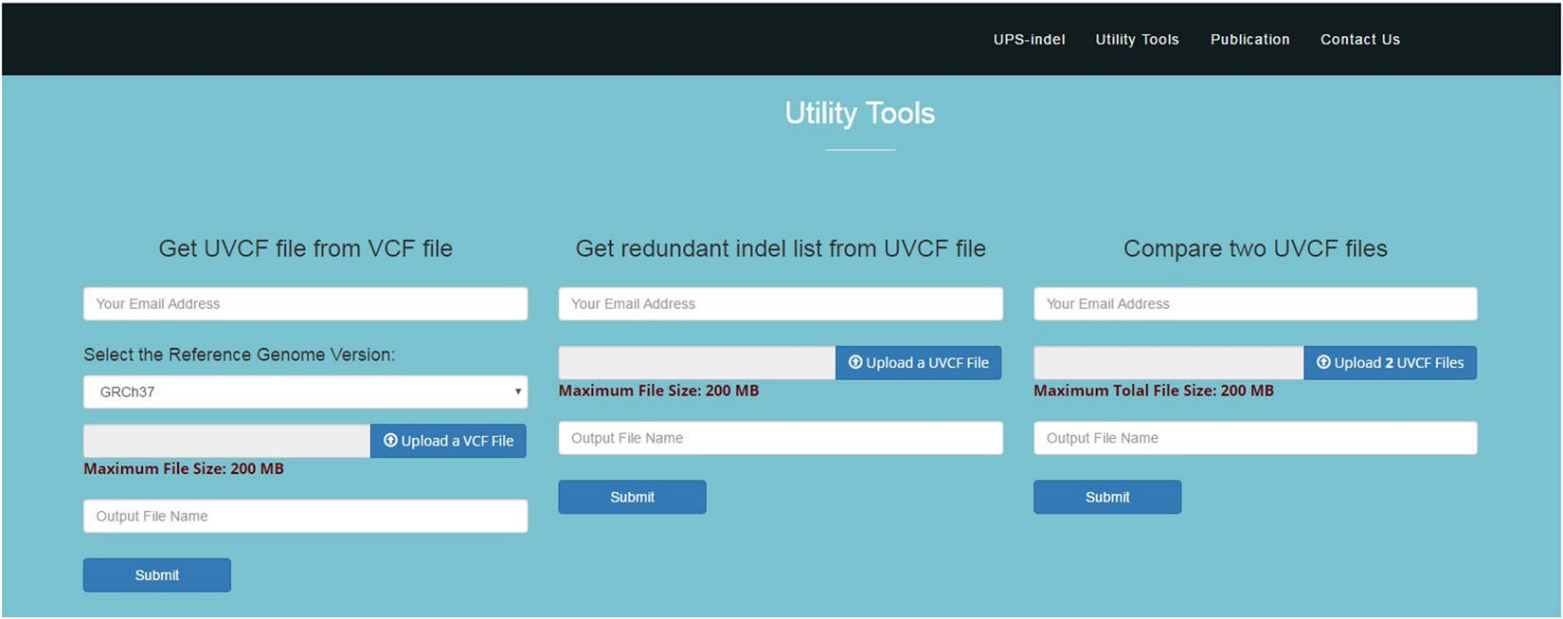
Main user interface of UPS-indel.

All of the above mentioned utilities of UPS-indel are also included in the web version available at http://bench.cs.vt.edu/ups-indel/ (Fig. 3).

UPS-indel is compared with other existing tools that also find equivalent indels through variant normalization. These tools include vt normalize (version 0.5)^2^, BCFtools (version 1.3)^8^, and GATK LeftAlignAndTrimVariants (version 3.5)^9^. Like UPS-indel, all of these tools take a VCF file and the reference genome as input and produce the normalized position of the indels in the VCF file. Another tool Vindel^4^ also finds equivalent indels using a heuristic approach, but was not included in the comparison as it uses a flat file as input instead of a VCF file.

A VCF file of dbSNP (version 142, GRCh37p13) and the GRCh37 reference genome were used as the inputs to these tools. The VCF file contains both SNPs and indels, and VCFtools^14^ (Version 0.1.14) is used to extract indels from the VCF file. The comparison was extended to the COSMIC dataset as well.

There are other tools that could also be considered for comparison. Both VarMatch^3^ and RTGTools^15^ use a branch and bound algorithm to search for equivalent indels. They are not suitable for processing population-scale indel call sets such as dbSNP and COSMIC because densely packed indels in such datasets make the search space too large to be processed by a branch and bound algorithm^3^. READDI^16^ considers repeat-induced ambiguities as well as tool-induced inaccuracies while searching for equivalent deletions using the longest common extension algorithm. This tool is limited to finding deletions only, and hence not included in the comparison for the dbSNP and COSMIC datasets. Nevertheless, in this study a smaller dataset is used to compare UPS-indel with VarMatch (Version available on April 5, 2017), RTGTools (Version 3.7.1), and READDI (Version available on April 5, 2017).

## Results and Discussion

### Finding equivalent indels in the dbSNP dataset

The input VCF file contains about 8.9 million indels from the human genome. For this input, UPS-indel produces the UVCF file and the other three tools, vt normalize, BCFtools, and GATK LeftAlignAndTrimVariants, generate the normalized VCF file. These three tools perform left normalization of indels and output a left normalized representation. Therefore, for these three tools, two indels are equivalent if and only if they satisfy the following conditions:

1. Both indels are of the same type (insertion or deletion).
2. Both indels share the same pattern after normalization: [value of the REF column in the normalized VCF file – value of the ALT column in the normalized VCF file – value of the POS column in the normalized VCF file]. Note that one might think that considering the position should suffice, because after normalization, equivalent indels should have the same position in the VCF file. However, the example in Table 4 shows that indels rs371246544 and rs71724031 have the same normalized position but are not equivalent.

**Table 4:** An example explaining why considering only normalized position does not suffice for identifying redundant indels for vt normalize and BCFtools.

#CHRM	POS	ID	REF	ALT
**VCF Entry for input**				
1	39549110	rs371246544	AT	ACATAC
1	39549111	rs71724031	T	TAC
**Entry in the normalized VCF**				
1	39549111	rs371246544	T	CATAC
1	39549111	rs71724031	T	TAC

$$\begin{array}{c}{\rm{The}}\,{\rm{comparison}}\,{\rm{is}}\,{\rm{based}}\,{\rm{on}}\,{\rm{the}}\,{\rm{criterion}}:{\rm{the}}\,{\rm{redundant}}\,{\rm{indel}}\,{\rm{ratio}}\,=\\ \frac{total\,number\,of\,redundant\,indels-total\,number\,of\,redundant\,indel\,groups}{total\,number\,of\,indels}\end{array}$$

where the numerator is the total number of redundant indels reported since only one indel from each redundant indel group should be reported in the output and the remaining should be considered as redundant.

**Figure 4:**
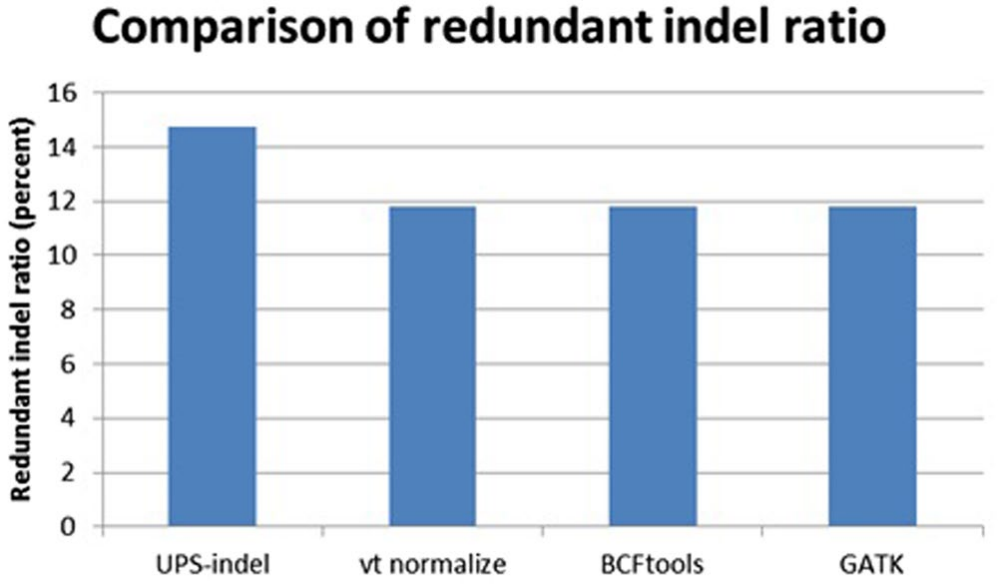
Comparison among the tools based on redundant indel ratio for the dbSNP dataset.

Figure 4 shows the comparison of the redundant indel ratios reported by UPS-indel, vt normalize, BCFtools, and GATK LeftAlignAndTrimVariants for indels in the dbSNP dataset. For the entire human genome, UPS-indel identified ~15% redundant indels (see Supplementary Table 3 and Supplementary Figure 1 for chromosome-wise comparison), as compared to 11.82% by vt normalize, 11.82% by BCFtools, and 11.81% by GATK LeftAlignAndTrimVariants. At the chromosome level, UPS-indel identified about 3% more redundant indels than the other three tools.

**Figure 5:**
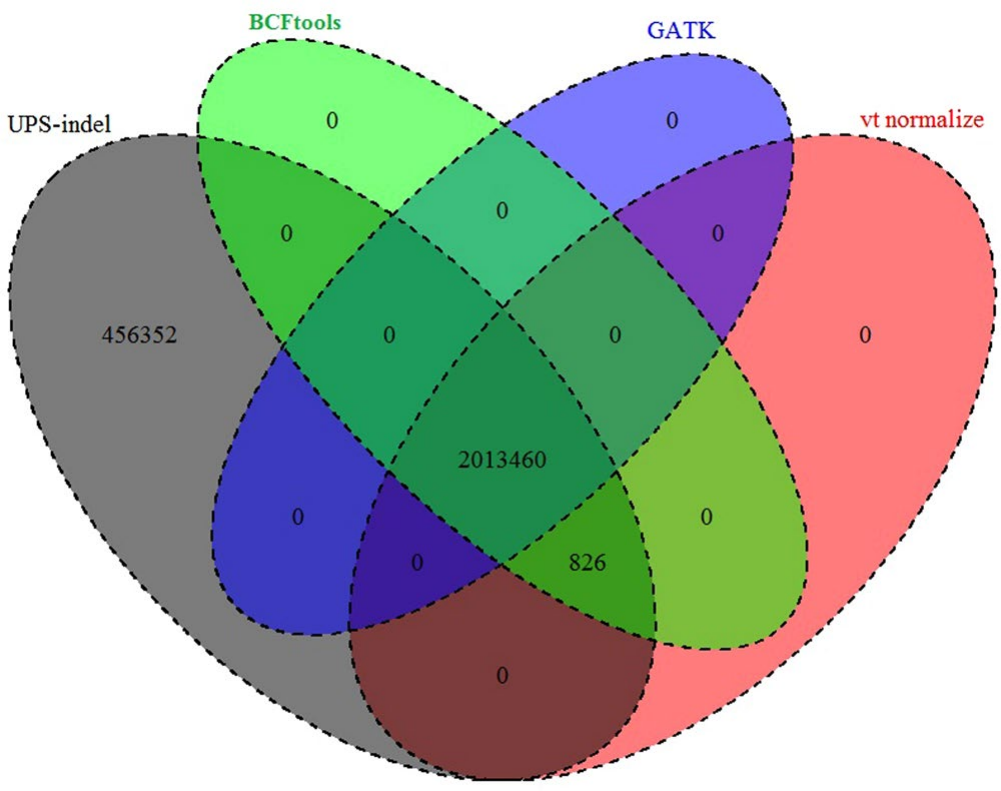
Venn diagram to compare the number of redundant indels detected by UPS-indel and other tools. (Venn Diagrams are generated using the R package VennDiagram^25^.)

Examining the sets of redundant indels detected by UPS-indel and the other tools shows that vt normalize and BCFtools produce exactly the same results for all chromosomes. Moreover, all the redundant indels detected by vt normalize, BCFtools, and GATK LeftAlignAndTrimVariants are also detected by UPS-indel, as shown in Fig. 5. Further, for all chromosomes, UPS-indel identified a total of 456,352 more redundant indels than the other tools. As proved in the methods, UPS-indel identifies all the redundant indels, the comparison result shows that the other three tools are not exhaustive in finding all the redundant indels.

Why are several indels found as redundant by UPS-indel but not by other tools? An investigation shows that these equivalent indels are missed by the other tools because, due to the computation time limit, they cannot exhaustively search every cyclic permutation at every feasible position as is done by UPS-indel. For example, long multiallelic indels are not considered by default for normalization. Had the tools considered these indels separately, they would have been able to find an equivalent indel located at a different position. For this situation, UPS-indel splits the VCF entry into multiple entries by default and considers each of the indels separately while finding redundant indels. Table 5 provides such an example.

**Table 5:** Example of multiallelic insertion type indels missed by other tools but detected as redundant by UPS-indel.

(A) VCF Entry					
#CHRM	POS	ID	REF	ALT	
1	724188	rs60022176	A	AATGGA, AATGGAATGGAATGGA, AATGGAATGGG	
**(B) UVCF Entry**					
**#CHRM**	**POS**	**ID**	**REF**	**ALT**	**UPS-COORDINATE**
1	724188	rs60022176	A	AATGGA	+ AATGG[724138 − 724189]
1	724188	rs60022176	A	AATGGAATGGAATGGA	+ AATGGAATGGAATGG[724138 − 724189]
1	724188	rs60022176	A	AATGGAATGGG	+ ATGGAATGGG[724189 − 724189]
**(C) Redundant indels**					
**#CHRM**	**POS**	**ID**	**REF**	**ALT**	**UPS-COORDINATE**
1	724137	rs374587598	T	TAATGG	+ AATGG[724138 − 724189]
1	724188	rs60022176	A	AATGGA	+ AATGG[724138 − 724189]

For the indel shown in Table 5 (panel A), no normalization was done by vt normalize, BCFtools, or GATK LeftAlignAndTrimVariants. UPS-indel splits the entry into three indels and finds the UPS-coordinate for each of them separately (Table 5, panel B). Splitting the VCF entry and considering the indels separately, UPS-indel managed to find another indel equivalent to one of the indels (Table 5, panel C). Therefore UPS-indel reports indels with id rs374587598 and rs60022176 as redundant.

The example in Table 5 is for insertion; an example for deletion is illustrated in Table 6.

**Table 6:** Example of multiallelic deletion type indels missed by other tools but detected as redundant by UPS-indel.

VCF Entry					
#CHRM	POS	ID	REF	ALT	
1	7552657	rs376707888	GTG	G, GTGCA	
**UVCF Entry**					
#CHRM	POS	ID	REF	ALT	UPS-COORDINATE
1	7552657	rs376707888	GTG	G	−GT[7552657 − 7552658]
1	7552657	rs376707888	GTG	GTGCA	+ CA[7552658 − 7552658]
**Redundant indels**					
**#CHRM**	**POS**	**ID**	**REF**	**ALT**	**UPS-COORDINATE**
1	7552656	rs139294420	CGT	C	−GT[7552657 − 7552658]
1	7552657	rs376707888	GTG	G	−GT[7552657 − 7552658]

In addition to the scenario mentioned above, GATK LeftAlignAndTrimVariants does not normalize any of the multiallelic indels regardless of the size which is also mentioned in ref.^2^. Table 7 shows an example of this occurrence explaining why GATKLeftAlignAndTrimVariants finds fewer number of redundant indels than vt normalize and BCFtools.

**Table 7:** Example of a multiallelic indel that is normalized by vt normalize and BCFtools but not by GATKLeftAlignAndTrim.

VCF Entry for dbSNP				
#CHRM	POS	ID	REF	ALT
1	823905	rs397728418	AA	A, AAA
**VCF Entry for GATK LeftAlignAndTrimVariants**				
**#CHRM**	**POS**	**ID**	**REF**	**ALT**
1	823905	rs397728418	AA	A, AAA
**VCF Entry for vt normalize and BCFtools**				
**#CHRM**	**POS**	**ID**	**REF**	**ALT**
1	823903	rs397728418	GA	G, GAA

One might think that decomposing multiallelic indels into several biallelic indels produces the same results as UPS-indel for the normalization tools. To check this, the “decompose” utility of vt was used to perform a vertical decomposition of multiallelic indels into biallelic indels. Applying vt normalize to the decomposed indels could not find equivalent indels for complex variants, whereas UPS-indel is able to find the equivalent indels. Table 8 shows an example of this occurrence. Since vt normalize and BCFtools produce exactly the same results, these complex variants are missed by BCFtools as well.

**Table 8:** Example of a complex variant that is missed by vt normalize but detected as redundant by UPS-indel.

VCF Entry (A)					
#CHRM	POS	ID	REF	ALT	
1	2273131	rs369694942	GAAA	G	
1	2273140	rs373243812	AAAAA	AG	
**UVCF Entry (B)**					
**#CHRM**	**POS**	**ID**	**REF**	**ALT**	**UPS-COORDINATE**
1	2273131	rs369694942	GAAA	G	−AAA[2273132 - 2273147]
1	2273140	rs373243812	AAAAA	AG	−AAA[2273132 - 2273147]

In the example shown in Table 8, VCF entries for the indels with ids rs369694942 and rs373243812 remain the same in the input and the output for vt normalize (Panel A), i.e., no normalization is done. Here the second indel (rs373243812) is a complex variant containing both a SNP (A → G) and a deletion of length three (AAA), and is ignored by vt normalize. However, UPS-indel performs a horizontal decomposition of the complex variant to produce two separate entries (AA → AG and AAA →*) and finds the equivalent indel with id rs369694942 having a deletion of length three (AAA) in the UPS-coordinate 2273132 to 2273147 (Panel B).

As defined in ref.^17^, complex variants come in two forms: (1) MNP and (2) clumped indel. In MNP, (1) the lengths of the reference and alternate sequences are greater than one and (2) the nucleotides involved in the two sequences differ. In clumped indel, on the other hand, there is a clumping of nearby variants and the sequences need not involve different base pairs. Table 9 shows an example of MNP and clumped indel found in dbSNP (version 142).

**Table 9:** An example of the two types of complex variant.

#CHRM	POS	ID	REF	ALT	Type
1	565003	rs386627398	ATAT	AAAC	MNP
1	884423	rs386627415	AGCA	AACAACAGCAAAG	Clumped Indel

UPS-indel decomposes complex variants based on the best predicted outcome using the Needleman-Wunsch algorithm. This approach was used by Li *et al*.^18^ to decompose complex variants into individual events.

### Finding equivalent indels in the COSMIC dataset

UPS-indel was used to find redundant indels in the COSMIC (Catalogue Of Somatic Mutations In Cancer) dataset, the world’s most comprehensive resource for exploring the impact of somatic mutations in human cancer^19^. With data collected for more than 2,500 human cancers, this archive describes millions of coding mutations, noncoding mutations, and other gene expression variants across the human genome.

**Figure 6:**
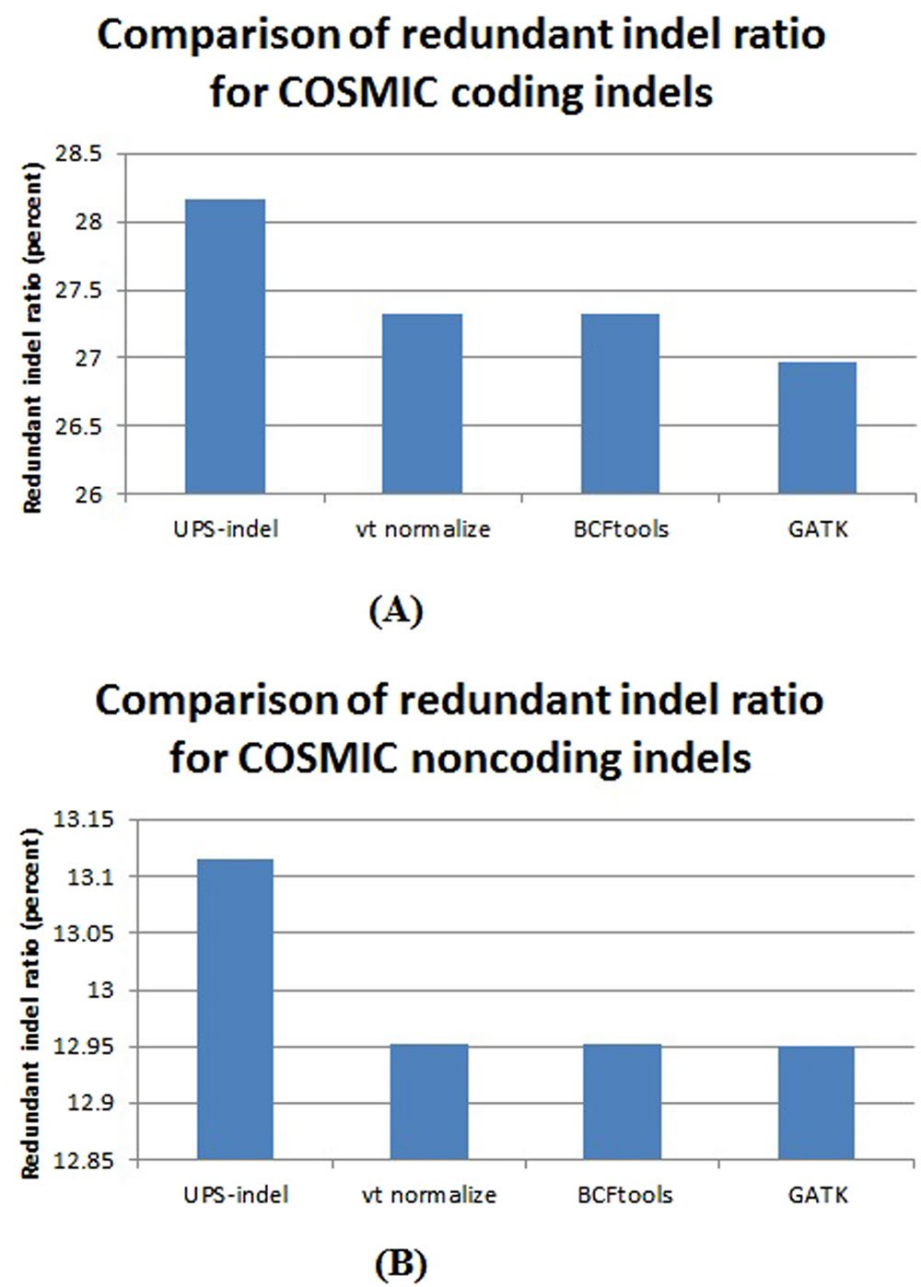
Comparison of redundant indel ratio for (**A**) COSMIC coding and (**B**) COSMIC noncoding indels.

For all chromosomes in the COSMIC dataset, UPS-indel identified 28.17% and 13.11% redundant indels in the COSMIC coding and noncoding indel datasets, respectively, which are higher than the redundant indel ratios reported by the other tools. Figure 6 shows the comparison of the redundant indel ratios reported by UPS-indel, vt normalize, BCFtools, and GATK LeftAlignAndTrimVariants for both the COSMIC coding and noncoding datasets. Comparisons for chromosome-wise redundant indel ratios among the tools are given in Supplementary Materials (See Table 4 and Fig. 2 for COSMIC coding and Table 5 and Fig. 3 for noncoding indels).

**Figure 7:**
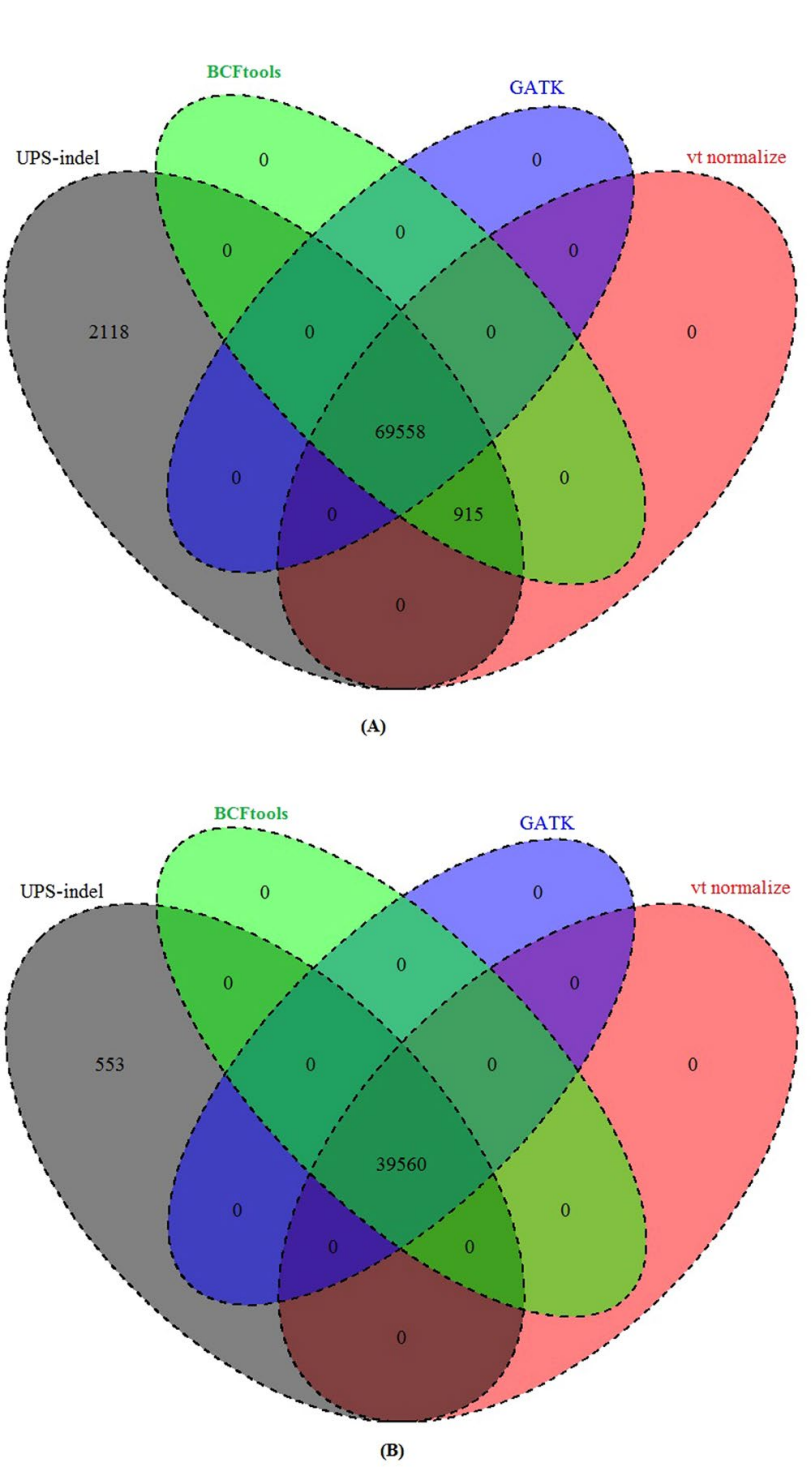
Venn diagram to compare the number of redundant indels detected by UPS-indel and other tools in (**A**) COSMIC coding and (**B**) COSMIC noncoding indel datasets.

Similarly, examining the sets of redundant indels identified by the tools, Fig. 7 shows that for both the COSMIC coding and noncoding indels, UPS-indel identified all the redundant indels detected by the other tools. In addition to that, for the whole genome, 2,118 (Fig. 6A) and 553 (Fig. 6B) unique redundant indels for COSMIC coding and noncoding indels, respectively, are detected by UPS-indel but missed by other tools.

As for dbSNP, the reason why some COSMIC coding and noncoding indels were considered as redundant by UPS-indel but missed by other tools is that, in the normalized VCF for these other tools, redundant indels must contain the same pattern: [value of the REF column in the normalized VCF file – value of the ALT column in the normalized VCF file – value of the POS column in the normalized VCF file]. The reason for this pattern match restriction was given earlier. In Table 10, all tools except UPS-indel missed the indel with id COSM5068028 in the redundant indel group consisting of indels with id COSM3732389 and id COSM5348791, because of not having the same pattern. Therefore it might be assumed that only normalized position should be considered to group them together. However, then the indel with id COSM3685916 would be placed in the same group, although it is a deletion type indel whereas the others are insertion type indels, and also the resultant sequences are different. UPS-indel groups the indels correctly by placing indels with ids COSM5068028, COSM3732389, and COSM5348791 in the same redundant indel group as they have the same base pair inserted, have the same region of equivalence, and also are of the same indel type.

**Table 10:** Example of COSMIC indel that is missed by other tools but detected as redundant by UPS-indel.

VCF Entry for COSMIC					
#CHRM	POS	ID	REF	ALT	
1	150917623	COSM5068028	TG	TGG	
1	150917623	COSM3732389	T	TG	
1	150917623	COSM3685916	TG	T	
1	150917624	COSM5348791	G	GG	
**VCF Entry for other tools after normalization**					
**#CHRM**	**POS**	**ID**	**REF**	**ALT**	
1	150917623	COSM5068028	TG	TGG	
1	150917623	COSM3732389	T	TG	
1	150917623	COSM3685916	TG	T	
1	150917623	COSM5348791	T	TG	
**UVCF Entry for UPS-indel**					
**#CHRM**	**POS**	**ID**	**REF**	**ALT**	**UPS-COORDINATE**
1	150917623	COSM5068028	TG	TGG	+ G[150917624 - 150917632]
1	150917623	COSM3732389	T	TG	+ G[150917624 - 150917632]
1	150917623	COSM3685916	TG	T	−G[150917624 - 150917631]
1	150917624	COSM5348791	G	GG	+ G[150917624 - 150917632]

GATKLeftAlignAndTrimVariants found fewer redundant indels than other tools because GATKLeftAlignAndTrimVariants does not consider very large indels for normalization. For example, the indels with ids COSM5196837 and COSM5066846, which are deletions of length 371 bps and 222 bps, respectively, are not considered by GATKLeftAlignAndTrimVariants for normalization. The reason is that GATK LeftAlignAndTrimVariants uses 200 bps as the default size of the sliding window on the reference (the parameter–reference_window_stop) while left aligning the alleles which is smaller than the length of the missed deletions.

These tools are also compared in terms of average running time taken to process the whole genome VCF file of dbSNP (version 142, size 16.7GB) for normalization (by vt normalize, BCFtools, and GATKLeftAlignAndTrimVariants) or for generating the UPS-coordinate (by UPS-indel). All tools were run on a desktop computer having an Intel Core i7-2600 CPU with eight cores (at 3.40 GHz) and 16GB of RAM. Among these tools vt normalize is the fastest, taking 12 minutes 27 seconds, followed by BCFtools (12 minutes 35 seconds), UPS-indel (17 minutes 10 seconds), and GATK LeftAlignAndTrimVariants (21 minutes 55 seconds). Since UPS-indel searches for equivalent indels exhaustively and is theoretically rigorous, the computation time is a little higher than that for other heuristic normalization tools such as vt normalize and BCFtools.

### Evaluating UPS-indel’s performance in comparing different indel call sets

In genomic research related to indel calling, an important step in downstream analysis is to compare multiple indel call sets for (1) generating a highly accurate benchmark indel call set by taking the intersection of multiple call sets as done by Zook *et al*.^12^ for the sample NA12878, (2) merging the call sets of different indel callers in a consensus caller as done by Trubetskoy *et al*.^13^ for exome data, and (3) evaluating the accuracy of a newly proposed indel calling tool by comparing its indel call set with the benchmark call set. Comparing different indel call sets is also a common step in studies comparing the performance of different indel callers as done in refs^5,20,21^. Different indel callers having different representations of the same indel complicates the comparison of different indel call sets. In addition to strict matching of indels, as mentioned earlier, a naïve but previously commonly used approach to compare multiple indel calling results is based on a simple distance criterion, that is, indels are considered to be equivalent if they are within a distance threshold (e.g., ± 5 bp or ± 25 bp). For example, the original 1000 Genomes project used ± 25 bp to compare multiple indel calling results^6^. To illustrate the advantage of using a UVCF file instead of a distance criterion or normalized VCF for comparing multiple VCF files, the alignment file for chromosome 11 of a single sample (HG00851) was picked up from the 1000 Genomes project and five indel callers: Dindel^22^ (Version 1.0.1), GATK Unified Genotyper^9^ (Version 3.4), GATK Haplotype Caller (Version 3.4), Platypus^23^ (Version 0.7.9.1), and Pindel^24^ (Version 0.2.5) were used to produce VCF files for indels. The resultant VCF files were compared to determine the number of common indels from these five tools using three different approaches, namely a distance based approach, comparing the VCF files normalized by vt normalize and GATK LeftAlignAndTrimVariants, and comparing the UVCF files produced by UPS-indel. For the distance based approach, two indels are considered equivalent if (1) they belong to the same indel type (either both are insertion type or both are deletion type), (2) have the same base pairs inserted/deleted, and (3) are in close proximity (within ± 5 bps from each other). For the normalized VCF files and UVCF files, the same approach was used as discussed earlier for finding redundant indels.

First the VCF files produced by the five indel calling tools were compared to find overlap among them to determine the number of common indels using the distance based approach. In the second step, the VCF files of the five indel calling tools were normalized using vt normalize and GATK LeftAlignAndTrimVariants separately. For this sample, both normalization tools produced the same normalized VCF files. The normalized VCF files of five indel calling tools were compared to determine the common number of indels. Finally, UPS-indel was used to produce the UVCF files for the five indel calling tools and these UVCF files were compared to determine the common number of indels.

The result shows that the distance based approach found 584 indels in common from the five indel calling tools while 5,514 and 5,575 common indels were found by the normalized VCF and UPS-indel UVCF approaches, respectively. This demonstrates the better suitability of UPS-indel, compared to distance based or existing normalization based approaches, for comparing multiple VCF files. Further investigation revealed that indels that are missed by the normalization tools are complex variants that are skipped by normalization tools but processed by UPS-indel. Note that this small number (61) of common indels identified by UPS-indel, but missed by the normalization tools, is based on a single chromosome of a single sample only, and much better performance of UPS-indel would be expected for the whole genome, as observed for the dbSNP and COSMIC datasets.

As mentioned earlier, the tools VarMatch^3^, RTG Tools^15^, and READDI^16^ are also used for comparing indel call sets. However, VarMatch and RTG Tools, which use a branch and bound algorithm, are not suitable for population-scale indel call sets like dbSNP and COSMIC due to densely packed indels in those call sets^3^. READDI processes deletions only. These tools are compared with UPS-indel (using the deletion call set of Platypus containing 14,438 deletions for chromosome 11 of the above mentioned single sample from the 1000 Genomes project as the baseline) on the deletion call sets of Dindel, GATK Unified Genotyper, GATK Haplotype Caller, and Pindel as the query call set to check overlap with the baseline. Table 11 shows the comparison for finding the number of true positives.

**Table 11:** Comparison between VarMatch, RTG Tools, READDI, and UPS-indel based on the number of true positives found between the baseline and query call sets from chromosome 11 of an individual.

Variant Caller Name	VarMatch (EVQ mode)	RTG Tools	READDI	UPS-indel
Dindel	8,933	8,933	8,796	8,973
GATK Haplotype Caller	11,113	11,113	10,954	11,129
GATK Unified Genotyper	7,734	7,734	7,563	7,734
Pindel	6,507	9,893	9,524	9,836

Table 11 shows that in all cases except for Pindel, UPS-indel finds more common indels than the state-of-the-art tools when comparing multiple indel call sets. These tools are heuristic and therefore ignore indels that violate a particular heuristic criterion. For example, READDI searches for equivalent indels in an indel’s neighboring region defined by the neighborhood size, and RTG Tools uses a cutoff strategy when the search space is too large. UPS-indel, on the other hand, exhaustively searches for and finds all equivalent indels, thus finds more common indels than the aforementioned tools. Why there are true positives identified by RTG Tools but missed by UPS-indel is because the current version of UPS-indel doesn’t consider haplotypes formed by the combination of neighboring indels. Consider the following example in Table 12.

**Table 12:** A combination of variants identified by RTG Tools but missed by UPS-indel.

Reference	1	2	3	4	5	6	7	8
	T	G	C	C	G	C	T	A
Deletion D1: Delete CC from position 3 and 4	TGGCTA							
Deletion D2: Delete C from position 6	TGCCGTA							
Deletion D1D2 (Combination of D1 and D2): Delete CC from position 3 and 4, also delete C from position 6	TGGTA							
Deletion D3: GCCGC - > GG starting from position 2	TGGTA							

In the Table 12 example, D3 is equivalent to D1D2. UPS-indel doesn’t consider the combination of D1 and D2 but rather considers them separately. Therefore, UPS-indel is not able to discover the aforementioned equivalence of the resultant haplotypes. This explains why UPS-indel missed 57 out of 9,893 deletions (where the deletions in a combination were separated by at most 45 bps) in the Pindel call set that are identified as equivalent by RTG Tools as shown in Table 11. Nevertheless there are 103 indels that are found as true positive by UPS-indel but missed by RTG Tools.

## Conclusion

This paper describes UPS-indel, a user friendly tool that creates a universal positioning system called UPS-coordinates for all indels listed in a VCF file, and exhaustively finds all equivalent indels. The UPS-coordinate is a range of positions where all indels equivalent to a specific indel can occur. Since equivalent indels produce the same mutant sequence and thus have the same biological effect, reporting them as separate indels causes data redundancy and may artificially inflate the statistics of indel variations. Under the proposed universal positioning system, all equivalent indels have the same UPS-coordinate which avoids possible annotation ambiguity. Therefore, by checking the UPS-coordinate, one can easily filter out redundant indels from variant databases. UPS-indel is robust enough to handle complex variants and is able to detect more redundant indels than the currently existing approaches. UPS-indel could be widely used for easy and accurate systematic comparison of indels generated by different indel calling programs or deposited in databases. By eliminating the indel redundancy issue, this work offers the community the proposed universal positioning system to represent indels (so as to avoid ambiguity), which can greatly improve various downstream genomic analyses related to indels.

### Availability of data and materials

The latest version of dbSNP VCF file can be found here: ftp://ftp.ncbi.nlm.nih.gov/snp/organisms/human_9606/VCF/. VCF file for the COSMIC coding mutation is available at http://grch37-cancer.sanger.ac.uk/cosmic/files?data=/files/grch37/cosmic/v78/CosmicCodingMuts.vcf.gz and noncoding mutation dataset is available at http://grch37-cancer.sanger.ac.uk/cosmic/files?data=/files/grch37/cosmic/v78/CosmicNonCodingVariants.vcf.gz. All of these VCF files contain SNPs, Indels, and other types of genetic variants. To extract only indels, we used VCFtools which is available at http://vcftools.sourceforge.net/. The command line version of UPS-indel is available at https://github.com/shabbir005/ups-indel with the instruction of how to install and use UPS-indel.

## Electronic supplementary material

Supplementary Materials
