# Supplementary material for: UPS-indel: a Universal Positioning System for Indels
Source: Sci Rep. 2017 Oct 26;7:14106. doi: 10.1038/s41598-017-14400-1 (PMC5658412; doi:10.1038/s41598-017-14400-1)
Supplement: Supplementary file 1 — Supplementary Materials [file 41598_2017_14400_MOESM1_ESM.pdf]

Supplementary Material

UPS-indel: a Universal Positioning System for Indels

Mohammad Shabbir Hasan[1], Xiaowei Wu[2], Layne T. Watson[1,3,4], and Liqing Zhang[1,*]

[1]Department of Computer Science, Virginia Tech, Blacksburg, VA 24061, USA.

[2]Department of Statistics, Virginia Tech, Blacksburg, VA 24061, USA.

[3]Department of Mathematics, Virginia Tech, Blacksburg, VA 24061, USA.

[4]Department of Aerospace and Ocean Engineering, Virginia Tech, Blacksburg, VA 24061, USA.

* Corresponding author: lqzhang@vt.edu

Supplementary Table 1: Example of redundant indels in dbSNP.

**Deletion**	
Reference Sequence	TTTGAAAAAAAAAAA
rs34434241	TTTG[A/-]AAAAAAAAAA
rs34174354	TTTGA[A/-]AAAAAAAAA
**Insertion**	
Reference Sequence	CAACCTAATGACTCCTT
rs200449532	CAAC[CTTT/+]CTAATGACTCCTT
rs4010175	CAACCT[TTCT/+]AATGACTCCTT

Supplementary Table 2: Example of equivalent deletions.

Reference sequence	ATAATGCCTGCCTGAAC
Case 1	ATAA[TGCC/-]TGCCTGAAC
Case 2	ATAAT[GCCT/-]GCCTGAAC
Case 3	ATAATG[CCTG/-]CCTGAAC
Case 4	ATAATGC[CTGC/-]CTGAAC
Case 5	ATAATGCC[TGCC/-]TGAAC
Case 6	ATAATGCCT[GCCT/-]GAAC

Case 7	ATAATGCCTG[CCTG/-]AAC
Mismatch	ATAATGCCTGC<u>***CTGA***</u>AC

Supplementary Table 3: Redundant indel ratio of UPS-indel, vt normalize, BCFtools and GATK LeftAlignAndTrimVariants.

**Chr Num**	**Total Indels**	**UPS-indel**		**vt normalize**		**BCFtools**		**GATK LeftAlignAndTrimVariants**	
		**Total redundant Indels**	**Redundant Indel Ratio*100**	**Total redundant Indels**	**Redundant Indel Ratio*100**	**Total redundant Indels**	**Redundant Indel Ratio*100**	**Total redundant Indels**	**Redundant Indel Ratio*100**
1	700390	105969	15.129999	85013	12.13795171	85013	12.13795171	84978	12.1329545
2	738109	109274	14.80458848	88014	11.92425509	88014	11.92425509	87981	11.91978421
3	619804	92047	14.85098515	73705	11.89166253	73705	11.89166253	73680	11.88762899
4	610352	90592	14.84258264	72321	11.84906415	72321	11.84906415	72293	11.84447663
5	553533	81877	14.7917107	65540	11.84030582	65540	11.84030582	65513	11.83542806
6	551502	83866	15.20683515	67234	12.19107093	67234	12.19107093	67209	12.18653785
7	502332	73054	14.54297158	58573	11.66021675	58573	11.66021675	58543	11.6542446
8	445604	64725	14.52522868	51822	11.62960835	51822	11.62960835	51800	11.62467123
9	367764	53863	14.64607738	43030	11.70043832	43030	11.70043832	43008	11.69445623
10	423800	65509	15.45752714	52382	12.36007551	52382	12.36007551	52356	12.35394054
11	415214	62539	15.06187171	50030	12.04920836	50030	12.04920836	50018	12.04631828
12	430538	64750	15.03932289	51511	11.964333	51511	11.964333	51491	11.95968765
13	316901	47490	14.98575265	37667	11.88604643	37667	11.88604643	37653	11.88162865
14	286524	43296	15.11077606	34345	11.98677947	34345	11.98677947	34327	11.98049727
15	263981	39720	15.04653744	31568	11.9584364	31568	11.9584364	31558	11.95464825
16	263863	38221	14.48516844	30190	11.44154353	30190	11.44154353	30177	11.43661673
17	265708	40554	15.26261912	32588	12.26459121	32588	12.26459121	32578	12.26082768
18	242978	36469	15.00917779	28971	11.9233017	28971	11.9233017	28964	11.92042078
19	216888	33062	15.24381247	26271	12.11270333	26271	12.11270333	26253	12.10440412
20	198594	30940	15.57952405	24884	12.53008651	24884	12.53008651	24879	12.52756881
21	131149	20757	15.82703642	16651	12.69624625	16651	12.69624625	16645	12.69167131
22	124352	18862	15.16823212	15164	12.19441585	15164	12.19441585	15159	12.19039501
X	213877	28103	13.1397953	22570	10.55279436	22570	10.55279436	22567	10.55139169
Y	24459	2586	10.57279529	2175	8.892432234	2175	8.892432234	2175	8.892432234

Supplementary Table 4: Redundant indel ratio of UPS-indel, vt normalize, BCFtools, and GATK LeftAlignAndTrimVariants for COSMIC coding indel dataset.

**Chr Num**	**Total Indels**	**UPS-indel**		**vt normalize**		**BCFtools**		**GATK LeftAlignAndTrimVariants**	
		**Total redundant Indels**	**Redundant Indel Ratio*100**	**Total redundant Indels**	**Redundant Indel Ratio*100**	**Total redundant Indels**	**Redundant Indel Ratio*100**	**Total redundant Indels**	**Redundant Indel Ratio*100**
1	14528	3779	26.0118392	3710	25.5368943	3710	25.5368943	3668	25.2477974
2	10621	3267	30.7598155	3228	30.3926184	3228	30.3926184	3180	29.9406836

3	9517	2657	27.9184617	2509	26.3633498	2509	26.3633498	2449	25.732899
4	6572	1761	26.795496	1642	24.9847839	1642	24.9847839	1628	24.771759
5	8197	2420	29.5229962	2262	27.5954618	2262	27.5954618	2177	26.558497
6	7493	2183	29.1338583	2152	28.7201388	2152	28.7201388	2134	28.4799146
7	7263	2114	29.1064298	2009	27.6607462	2009	27.6607462	1982	27.288999
8	4992	1428	28.6057692	1410	28.2451923	1410	28.2451923	1397	27.9847756
9	6512	2104	32.3095823	2022	31.0503686	2022	31.0503686	1996	30.6511057
10	6284	1884	29.9809039	1821	28.9783577	1821	28.9783577	1790	28.4850414
11	8177	1986	24.2876361	1896	23.1869879	1896	23.1869879	1858	22.7222698
12	7793	2124	27.2552291	2096	26.8959322	2096	26.8959322	2077	26.6521237
13	3116	916	29.3966624	901	28.915276	901	28.915276	895	28.7227214
14	4340	1121	25.8294931	1101	25.3686636	1101	25.3686636	1095	25.2304147
15	4009	1077	26.8645548	1073	26.7647792	1073	26.7647792	1057	26.3656772
16	5487	1273	23.2002916	1250	22.781119	1250	22.781119	1235	22.5077456
17	11175	3888	34.7919463	3636	32.5369128	3636	32.5369128	3599	32.2058166
18	2132	554	25.9849906	548	25.7035647	548	25.7035647	541	25.3752345
19	9243	2259	24.4401168	2173	23.509683	2173	23.509683	2113	22.8605431
20	3312	869	26.2379227	852	25.7246377	852	25.7246377	841	25.3925121
21	1525	364	23.8688525	347	22.7540984	347	22.7540984	342	22.4262295
22	3221	750	23.2846942	733	22.7569078	733	22.7569078	728	22.6016765
X	6083	2020	33.207299	1959	32.2045044	1959	32.2045044	1931	31.7442052
Y	51	19	37.254902	19	37.254902	19	37.254902	19	37.254902

Supplementary Table 5: Redundant indel ratio of UPS-indel, vt normalize, BCFtools, and GATK LeftAlignAndTrimVariants for COSMIC noncoding indel dataset.

**Chr Num**	**Total Indels**	**UPS-indel**		**vt normalize**		**BCFtools**		**GATK LeftAlignAndTrimVariants**	
		**Total redundant Indels**	**Redundant Indel Ratio*100**	**Total redundant Indels**	**Redundant Indel Ratio*100**	**Total redundant Indels**	**Redundant Indel Ratio*100**	**Total redundant Indels**	**Redundant Indel Ratio*100**
1	16553	2123	12.8254697	2083	12.5838217	2083	12.5838217	2083	12.5838217
2	16617	1875	11.2836252	1850	11.1331769	1850	11.1331769	1850	11.1331769
3	12648	1444	11.4168248	1423	11.2507906	1423	11.2507906	1423	11.2507906
4	12146	1066	8.776552	1052	8.6612877	1052	8.6612877	1051	8.6530545
5	11770	1130	9.6006797	1112	9.4477485	1112	9.4477485	1112	9.4477485
6	11169	1324	11.8542394	1309	11.7199391	1309	11.7199391	1309	11.7199391
7	11950	1602	13.4058577	1588	13.2887029	1588	13.2887029	1588	13.2887029
8	9890	1128	11.4054601	1115	11.2740142	1115	11.2740142	1115	11.2740142
9	8469	1288	15.2084071	1278	15.0903294	1278	15.0903294	1278	15.0903294
10	8872	943	10.628945	922	10.3922453	922	10.3922453	922	10.3922453
11	9036	1312	14.519699	1305	14.4422311	1305	14.4422311	1305	14.4422311
12	9646	1256	13.0209413	1239	12.8447025	1239	12.8447025	1239	12.8447025
13	5906	485	8.2119878	475	8.0426685	475	8.0426685	475	8.0426685
14	6244	701	11.2267777	693	11.0986547	693	11.0986547	693	11.0986547
15	5828	714	12.2512011	702	12.0452986	702	12.0452986	702	12.0452986
16	6234	870	13.9557267	860	13.795316	860	13.795316	860	13.795316

17	6999	1073	15.3307615	1063	15.187884	1063	15.187884	1063	15.187884
18	4904	482	9.8287113	475	9.6859706	475	9.6859706	475	9.6859706
19	6101	1322	21.6685789	1300	21.3079823	1300	21.3079823	1300	21.3079823
20	4349	481	11.0600138	475	10.922051	475	10.922051	475	10.922051
21	3070	565	18.4039088	560	18.2410423	560	18.2410423	560	18.2410423
22	3109	558	17.9478932	546	17.561917	546	17.561917	546	17.561917
X	6831	870	12.7360562	863	12.6335822	863	12.6335822	863	12.6335822
Y	561	102	18.1818182	102	18.1818182	102	18.1818182	102	18.1818182

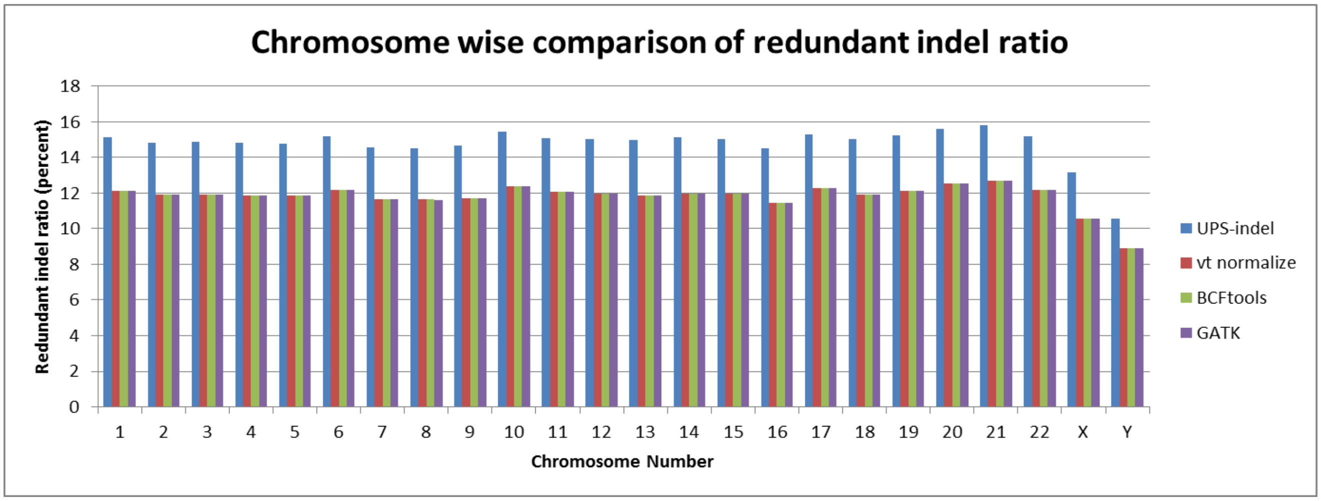

Supplementary Figure 1: Chromosome wise comparison of redundant indel ratio among UPS-indel, vt normalize, BCFtools, and GATK LeftAlignAndTrimVariants for the dbSNP dataset.

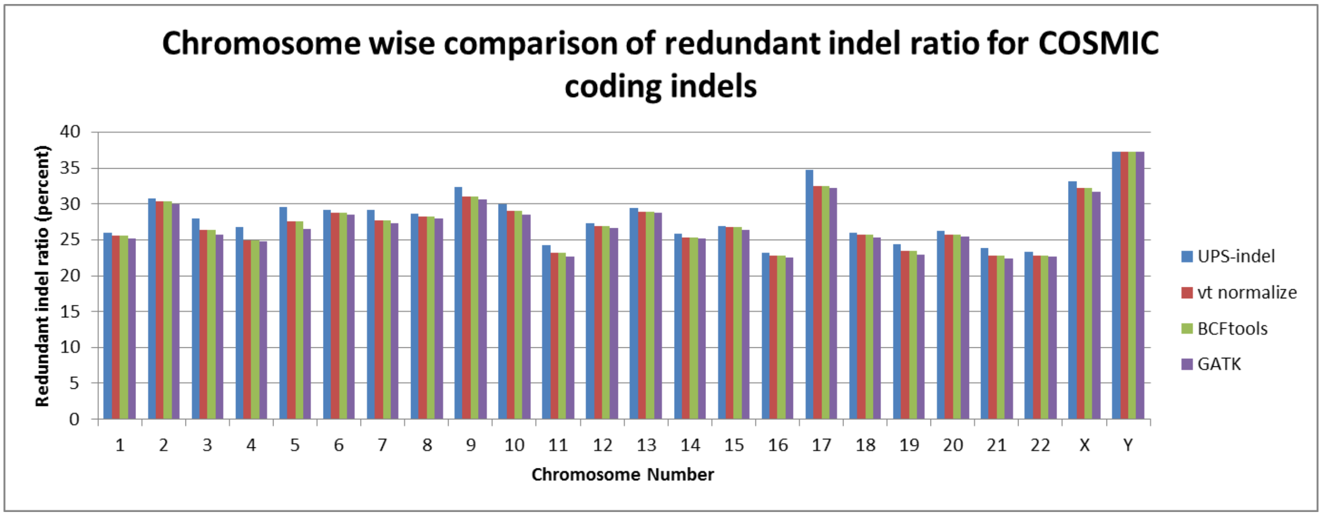

Supplementary Figure 2: Chromosome wise comparison of redundant indel ratio among UPS-indel, vt normalize, BCFtools, and GATK LeftAlignAndTrimVariants for COSMIC coding indels.

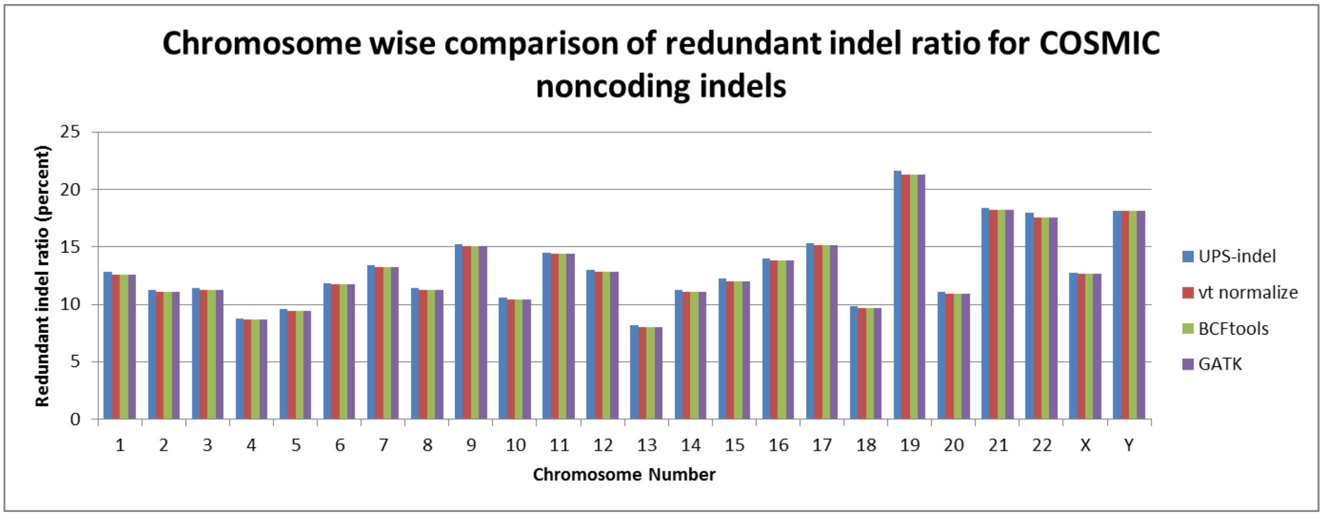

Supplementary Figure 3: Chromosome wise comparison of redundant indel ratio among UPS-indel, vt normalize, BCFtools, and GATK LeftAlignAndTrimVariants for COSMIC noncoding indels.
